# Public Knowledge, Attitudes, and Practices toward Seasonal Influenza Vaccine in Saudi Arabia: A Cross-Sectional Study

**DOI:** 10.3390/ijerph18020479

**Published:** 2021-01-08

**Authors:** Ibrahim A. Sales, Wajid Syed, Majed F. Almutairi, Yazed Al Ruthia

**Affiliations:** 1 Department of Clinical Pharmacy, College of Pharmacy, King Saud University, P.O. Box 2454, Riyadh 11451, Saudi Arabia; isales@ksu.edu.sa (I.A.S.); wali@ksu.edu.sa (W.S.); m.f.almutairi837@gmail.com (M.F.A.); 2 Pharmacoeconomics Research Unit, College of Pharmacy, King Saud University, P.O. Box 2454, Riyadh 11451, Saudi Arabia

**Keywords:** influenza, knowledge, attitudes, practices, vaccination

## Abstract

**Objectives:** Increasing national influenza vaccination rates continues to be a challenge for Saudi Arabia. Therefore, the purpose of this study was to explore the Saudi public perceptions toward seasonal influenza vaccination and their association with the rate of vaccination. **Methods:** Individuals aged 15 years and older were surveyed about their knowledge, attitudes, and practices toward the seasonal influenza vaccine using a previously developed and validated 19-item online questionnaire. The impact of the participants’ perceptions toward the seasonal influenza vaccine on their past influenza vaccination history was assessed using multiple linear regressions. **Results:** The rate of regular vaccination among the 790 surveyed participants was 12.65%, and those who were aged <24 years had the highest rate (57%). The vast majority of the participants with chronic diseases (>90%) reported irregular vaccination histories against seasonal influenza. Participants who believed that the influenza vaccine is safe (β = 3.27; 95% CI: 2.067 to 5.171; *p* <0.001), efficacious (β = 2.87; 95% CI: 1.834 to 4.498; *p* <0.001), should be given during a specific time in the year (β = 1.821; 95% CI: 1.188 to 2.789; *p* = 0.0059), and were aware of their need to get vaccinated against the seasonal influenza (β = 2.781; 95% CI: 1.254 to 6.188; *p* = 0.0119) were more likely to have received the vaccine. **Conclusion:** The findings of this study suggest that the rate of seasonal influenza vaccination is low among the Saudi population, which necessitates the launching of public awareness campaigns about the importance of the seasonal influenza vaccine.

## 1. Introduction

Three to 5 million cases of severe seasonal influenza, and nearly 650,000 related deaths globally, are being reported annually [1]. The World Health Organization (WHO) Global Vaccine Action Plan (GVAP), which was endorsed in 2012 by the World Health Assembly, aims to increase the rate of seasonal influenza vaccination to reduce the transmission of this global epidemic [2]. However, several challenges face the GVAP goal to increase the voluntary vaccination against influenza, particularly vaccine hesitancy. This has been faced with almost all vaccines before, such as diphtheria-tetanus-pertussis and hemophilus influenza [2,3,4,5,6,7,8,9]. Even in light of the recent COVID-19 pandemic, many remain skeptical and intend to abstain from voluntary vaccinations based upon myths, misinformation, and/or safety concerns [10,11,12,13,14]. Low vaccination rates against the seasonal influenza can exacerbate the situation and result in the spread of infectious diseases, such as the seasonal influenza, both locally and globally. This is especially worrisome in the case of Saudi Arabia where Muslims from all over the world visit to perform their religious duties of Umrah (minor pilgrimage) on a daily basis and the holy pilgrimage or Hajj (major pilgrimage) every year [15,16,17,18]. The interaction among such a wide variety of people from various socioeconomic classes, countries of differing development stages, and high influenza positivity rates increase the likelihood of exposure, infection, and eventually transmission of the disease domestically and internationally [19,20]. Moreover, Hashem et al. emphasized the potential hazards during these gatherings by mentioning that this is a breeding ground for new or highly pathogenic strains and/or resistant viruses [21]. Moreover, it was reported that the prevalence of influenza-like illnesses during the holy pilgrimage in Saudi Arabia ranged from 8 to 78.2% based on a systematic review conducted by Benkouiten et al. [22]. Additionally, administration of the seasonal influenza vaccine was believed to lower the rate of infection from 10% to 0% among the domestic pilgrims who were vaccinated against influenza, and from 14 to 7% among vaccinated pilgrims from the United Kingdom [22].

Despite the importance and urgency of implementing preventive protocols and taking precautionary measures, ultimately, the success depends on people’s adherence to these protocols and measures. Currently, all international visitors to Saudi Arabia who wish to perform Hajj or Umrah are advised to vaccinate against the seasonal influenza by the Saudi Ministry of Health [23,24]. However, this remains a recommendation and no domestic or international visitor to the holy sites is prohibited from performing Hajj or Umrah if he/she did not vaccinate against the seasonal influenza. Therefore, many health researchers and local professional medical associations are calling for making it a requirement for anyone who wishes to visit the Islamic holy sites [23]. This prompted the Saudi Thoracic Society to draft and publish guidelines for influenza vaccinations in 2015 including a special emphasis on the major and minor holy pilgrimages [24]. Locally, since 2017, the Influenza Surveillance in Saudi Arabia (ISSA), under the supervision of the Ministry of Health, has been assigned with monitoring, analyzing, planning, and disseminating information at the governmental and public levels to prevent and manage the impact of the disease [25]. In addition, the Ministry of Health published guidelines for the management and prevention of seasonal influenza in the healthcare settings [26]. The Ministry of Health recommends that healthcare professionals and the public receive the influenza vaccination on a yearly basis. Furthermore, the vaccine is available free of charge to all Saudi citizens [27].

Exploring and understanding the different sociodemographic and medical factors that may hinder the voluntary vaccination against the seasonal influenza is crucial to formulate an effective national policy aimed at improving the vaccination rate against this epidemic as well as other epidemics and pandemics such as COVID-19. Therefore, the objective of this study was to explore the public attitudes, knowledge, and practices toward the seasonal influenza vaccine and their association with the participants’ past influenza vaccination histories.

## 2. Methods

This was an online, questionnaire-based (Appendix A), cross-sectional study that was conducted between August and December 2016. The questionnaire was disseminated using a Google^®^ form on different social media platforms, such as WhatsApp^®^ and Twitter^®^, among individuals aged 15 years and older using a convenience sampling technique. The questionnaire consisted of 19 items covering four different domains and was developed and validated by El Khoury and Salameh [28]. The first domain included sociodemographic questions including age, gender, marital status, income, educational level, insurance status, and employment. The second domain collected information related to the participants’ medical history of chronic diseases. The third domain collected the vaccination status including the date of the last vaccine received, in general, and the influenza vaccine in particular. The fourth domain of the questionnaire assessed the knowledge and attitudes toward the influenza vaccine. The questionnaire was translated to Arabic using the forward-backward method [29]. The content validity was assessed by the authors of the study. Pilot testing was conducted to assess the face validity and internal consistency of the questionnaire among 10 Arabic-speaking individuals. The questionnaire showed acceptable internal consistency with a Cronbach’s alpha of 0.7 [30]. The study protocol was approved by the research ethics committee of the College of Pharmacy at King Saud University in Saudi Arabia (Approval ID number 12/2016). The minimum sample size for this study was estimated to be 732 respondents based on an effect size of w = 0.15, α = 0.05, β = 0.1, and power of 0.9.

## 3. Statistical Analysis

Descriptive statistics using percentages, frequencies, and means were conducted. Multiple linear regressions were conducted to examine the impact of participants’ perceptions on their influenza vaccination history controlling for age, comorbidities, gender, educational level, and financial and employment status. Statistical significance was considered at α < 0.05. All statistical analyses were conducted using SAS^®^ version 9.4 (SAS Institute Inc., Cary, NC, USA).

## 4. Results

### 4.1. Participants

The questionnaire was sent to 7560 individuals via various social media platforms. The number of participants who completed the questionnaire was 790 respondents, which represented a 10.4% response rate. The majority of the participants (69%) were female and more than 50% of the respondents were under 24 years of age. Most participants (61.5%) had a university degree and 33.4% of the respondents completed a high school diploma. Only 100 participants (12.65%) reported receiving regular influenza vaccine every year and the majority of them (87%) were under 33 years of age. Most participants (74.5%) were unemployed and 47.8% reported having no medical insurance. The participants were mostly healthy; however, about 8.35% of them reported having respiratory diseases (e.g., asthma and chronic obstructive pulmonary disease), 2.15% reported having diabetes, 1.13% reported having cardiovascular disease, and only 0.63% reported having chronic kidney disease. No significant differences in the sociodemographic or medical characteristics were noticed between the participants who received the vaccine on a regular basis and those who did not (Table 1). Table 2 shows knowledge, attitudes, and practices related to the influenza vaccination.

### 4.2. Knowledge, Attitudes, and Practices Regarding the Influenza Vaccination

The majority of participants (83.2%) reported that they were aware of the influenza symptoms and more than two-thirds of them were aware of the severity of influenza. In addition, approximately two-thirds of participants were aware of the risks related to influenza. However, no significant differences were noted between vaccinated and unvaccinated groups (*p* > 0.05). A significantly higher percentage of regularly vaccinated participants (e.g., those who annually received the vaccination) were convinced of the necessity of administering the influenza vaccination as compared to the irregularly vaccinated group (e.g., those who either never received the vaccination or had an interrupted history of vaccination against the seasonal influenza) (*p* = 0.031). The majority of participants in both groups considered the influenza vaccine to be safe and effective.

Regularly vaccinated participants were significantly more likely to believe that the influenza vaccine should be taken at a specific time compared to their counterparts who had an interrupted vaccination history against seasonal influenza (*p* = 0.002). In addition, a significantly higher number of participants in the irregularly vaccinated group thought that the influenza vaccine is only indicated for children (*p* = 0.001) (Table 2). As shown in Table 3, it was determined that the most common source of information was the media (58.3%), followed by parent(s)/family (41.8%) and physicians (21%).

### 4.3. Multiple Linear Regression

Perceptions toward the seasonal influenza vaccine with regard to its safety and efficacy, the belief that the vaccine should be administered during a specific time in the year, and the awareness of the need to get vaccinated against the seasonal influenza were positively associated with a regular vaccination history, as shown in Table 4. No other factors were found to be significant.

## 5. Discussion

The results from this study found an overall vaccination rate of 12.6%. This vaccination rate is lower than the previous findings published among the general public, the military, pregnant women, and healthcare workers in Saudi Arabia; however, this study most closely resembles a recent study by Alqahtani et al. in terms of study design, sample size, and vaccination rates [31]. An inoculation rate of 15% was reported in their cross-sectional survey among a sample of 1105 residents of Saudi Arabia. This study included fewer participants; nevertheless, it contributes to the effort to further describe the current situation in Saudi Arabia and provides insightful details that may lead to increasing the national inoculation rates. In addition, it is likely that these studies may be closer in actuality to the true vaccination rates of the general public as opposed to contained environments such as hospitals and special populations [31].

Unfortunately, this inoculation rate is six times lower than the recommended uptake rate proposed by the WHO GVAP program. On an international level, countries such as the United States (US), the United Kingdom (UK) (UK-England, UK-Northern Ireland, UK-Scotland), Germany, France, Spain, and Italy have reported significantly higher uptake rates of 44.8%, (72.7%, 73.4%, 76.3%), 40.4%, 37.5%, 56.4%, and 48.6%, respectively [32,33]. Regionally, influenza vaccination rates in Saudi Arabia are lower than its neighboring countries. Alqahtani et al. reported an influenza uptake rate of 24% in Qatar, 22% in the United Arab Emirates, 18% in Bahrain, 17% in both Oman and Kuwait, and 15% in Saudi Arabia [31]. Awaidy et al. examined the influenza surveillance systems in the Middle East North Africa (MENA) region [34]. They concluded that, although most countries had surveillance monitoring in place, the collected data was not being used efficiently. Furthermore, Saudi Arabia was not included in the list of the countries that used the data to determine the disease burden.

Approximately 44% of the participants in the study were between 24–51 years of age. This is similar to the country demographics in Saudi Arabia. Nearly 50% of the population is aged 25–54 years. In addition, it has been reported that 50% of the local Umrah pilgrims are 30–49 years old [15]. Participants in this study had a high level of education. Approximately 62% had university degrees and 33% had at least graduated from high school. Alqahtani et al. reported that 9% of their study population who had received the influenza vaccination had continued their studies beyond secondary education and 7% had not [31]. Mayet et al. indicated that 64.3% of pregnant women who participated in an influenza vaccination survey had obtained a bachelor degree or higher [35]. Although it may seem intuitive that a higher level of education typically results in higher vaccination uptake, this theory has been inconsistent in the international literature. Higher education has been identified as a potential barrier toward vaccination in the US, China, Lebanon, Bangladesh, and Israel [36,37,38,39,40]. Therefore, it is not unusual that the vaccination rate in our study was low, although most participants were well-educated.

Furthermore, vaccination uptake was extremely poor among patients with cardiovascular disease, diabetes, respiratory disorders, and renal disease. These patients are more susceptible to worse outcomes of the influenza [41,42,43,44]. Alqahtani et al. reported suboptimal inoculation rates (21%) for high-risk patients with chronic disease states among the Arabian Gulf countries [31]. In the European Union (EU), a 75% vaccination coverage rate for high-risk groups is targeted [33]. Recent data indicates that among high-risk groups, uptake rates have reached approximately 44% [44]. Therefore, more emphasis must be placed upon vaccinating high-risk populations in Saudi Arabia from both governmental and healthcare professional perspectives.

The multiple linear regression results confirmed that positive views toward influenza vaccine safety and efficacy were associated with higher rates of vaccine uptake. Public health education and information regarding vaccines, such influenza vaccination recommendations for specific populations and the optimal time for the influenza vaccination, also motivated participants to become vaccinated. Furthermore, although the majority of pregnant women in Saudi Arabia believed that the influenza vaccine was not safe to be used in pregnancy, two studies that explored public perceptions of the seasonal influenza vaccine among healthy adults reported that only few respondents had concerns regarding the safety and efficacy of the vaccine [31,35,44]. Only 21% of respondents in this study reported that their physicians were the primary source of their information. The primary sources reported were the media and family. This finding calls on physicians to engage their patients in candid conversations about their perceptions and beliefs toward influenza vaccination.

In the general Saudi population, participants’ inoculation rates have varied widely from 15 to 44.53% in healthy adults, 17.8% among military personnel, and 18.1% in pregnant patients [31,35,44,45]. The most common barriers included unawareness of the vaccine, confidence in the body’s natural immunity, and concerns about safety in pregnancy [35,45]. Medical students reported a 20.7% influenza vaccination rate and cited the main barriers were that they did not consider themselves to be at risk for influenza and concerns about potential side effects of the vaccine [46]. Among healthcare workers, the rates of inoculation ranged from 34.4 to 88.3% [47,48]. Barriers included the belief that vaccination was not important, misconceptions that the vaccination itself can cause influenza, concerns about vaccine efficacy, and fears of contracting an illness. In another study among a group consisting of parents, adult patients, and healthcare workers, the most common reasons for vaccine refusal were being unconvinced of the efficacy of the vaccine, the belief that a healthy lifestyle alone prevents influenza, and concerns about potentially serious side effects [46]. Awaidy et al. identified additional barriers, such as lack of conviction and logistical barriers (e.g., equipment, time consumption, and infrastructure), among healthcare workers [34]. On the other hand, lack of awareness was the primary barrier among the public.

The study results would appear to indicate that there are no specific or clear differences that may indicate a reason why the participants are not taking the vaccination from the demographical data or the information collected regarding the knowledge, attitudes, and practices about the vaccination. A possible explanation may be the influence of social media. Approximately 60% of respondents disclosed that their primary source of information regarding vaccination was the media. It was reported in a 2018 survey by the Saudi Communications and Information Technology Commission that approximately 93% of males and 90% of females were active on social media networks in Saudi Arabia [49]. Furthermore, some social media outlets have been associated with promoting vaccine hesitancy and misinformation about vaccines and vaccination [9,13,50,51]. Several groups, organizations, and individuals have spread through social media in Saudi Arabia warnings and posts intended to dissuade others from partaking in these vaccinations [52]. However, Awaidy et al. reported that motivating factors toward taking the vaccination include their doctor’s recommendation to take the vaccination, free access to the vaccination, and increasing awareness among the public [34]. The study results indicated that one-fifth of participants considered their physicians as their primary source of information regarding vaccinations. This presents a golden opportunity for physicians to provide patients with unbiased advice about the influenza vaccine as well as other vaccinations.

Various solutions to address vaccine hesitancy have been published in the literature. Gagneur et al. described an approach involving motivational interviewing tailored to each patient’s particular needs and concerns [53]. Agrawal et al. suggest utilizing communication strategies such as the media and religious leaders, education and awareness programs, and addressing parents’ safety concerns [54]. Braun and O’Leary recommended using a presumptive method of communicating with parents, social marketing and increasing awareness of the vaccination rates in schools and childcare centers, and governmental mandates in order to counter vaccine hesitancy in pediatric patients [55]. Puri et al. recognized the challenges of social media and offered strategies to use this platform to spread correct information [56]. Possible solutions include facilitating direct communication between providers and patients, encouraging social media networks to prompt users to confirm the accuracy of their posts as well as the agency being diligent in identifying and flagging inappropriate and inaccurate information, and using patient narratives and/or celebrity endorsements. Other approaches include smartphone apps, digital gamification, electronic reminder systems, shared-decision making, and properly training and preparing healthcare practitioners to communicate with patients, address their concerns, and promote vaccination programs to increase vaccination knowledge and reduce the frequency of vaccine hesitancy [57,58,59,60].

### Limitations

Limitations of this study include the possibility of selection bias. The participation of the elderly and patients of lower socioeconomic statuses may have been limited due to lack of access to the platform and the recruitment method. There is a possibility of recall bias since the data was collected through a self-reported survey. In addition, there may have been demographic bias due to the disproportionate number of female, younger, and unemployed participants. However, approximately 40% of the Saudi population is under the age of 25 years and, since the influenza vaccination is available at no cost, unemployment is not a barrier. The response rate was low; however, the number of participants included in the study is modest in comparison to the previously published influenza inoculation rates in Saudi Arabia. Nevertheless, non-response bias cannot be excluded. Further, the collected data did not indicate which region in Saudi Arabia the participants reside in. Vaccination uptake and awareness rates may be variable across regions. Additionally, our study did not differentiate between the Saudi nationals and expatriates. Practices and beliefs between these populations may influence participants’ responses. Finally, the sample size was smaller than some of the most recently published local and international studies. Therefore, these limitations may affect the generalizability of study findings.

## 6. Conclusions

This study concluded that the rate of vaccination in Saudi Arabia is very low despite participants’ acceptable level of knowledge about the influenza vaccine. Increasing the influenza vaccination rate among the Saudi population is very important to ensure a homogeneous population-immunity level. Addressing influenza vaccine safety, efficacy, optimal vaccination administration times, patient-specific vaccination recommendations, encouraging effective physician communication and counseling, and utilizing social media platforms to disseminate information from authentic and reliable sources may be key target areas for increasing knowledge and awareness as well as combating incorrect information regarding the influenza vaccination. Moreover, healthcare professionals should be consistently reminded of the importance of screening and counseling patients regarding vaccinations. In addition, different public healthcare entities should capitalize on the opportunities to provide education and accurate information regarding the influenza vaccination in particular and vaccinations in general during this period of heightened awareness due to the COVID-19 pandemic. Future studies should incorporate various means of data collection, such as online surveys and face-to-face interviews. Also, patients should be recruited from multiple regions and settings throughout the country, and participant nationality should be determined.

## Figures and Tables

**Table 1:** Participant sociodemographic characteristics.

Characteristic	Regular Influenza Virus Vaccination (*N* = 100)	Irregular Influenza Virus Vaccination (*N* = 690)	*p*-Value
Gender			
Male	31 (31%)	209 (30.1%)	0.885
Female	69 (69%)	481 (69.3%)	
Age groups			
<24 years	57 (57)	375 (54.1)	
24–33 years	30 (30)	250 (36)	
34–51 years	10 (10)	60 (8.6)	
52–64 years	3 (3)	5 (0.7)	0.188
Education level			
No education	2 (2)	3 (0.4)	
Below high school	6 (6)	27 (3.9)	
High school	31 (31)	234 (33.9)	0.254
University degree	61(61)	426 (61.8)	
Employment			
Yes	31 (31)	170 (24.6)	
No	69 (69)	517 (74.9)	0.180
Health Insurance			
None	44 (44)	333 (48.2)	
Governmental	39 (39)	229 (33.1)	
Private	17 (17)	128 (18.5)	0.518
Financial situation			
Comfortable	26 (26)	208 (30.1)	
Manageable	61 (61)	401 (58.1)	
Difficult	13 (13)	80 (11.5)	0.715
Chronic diseases			
Cardiovascular disease			
Yes	2 (2%)	7 (1.1%)	
No	98 (98%)	683 (98.9%)	0.319
Diabetes			
Yes	2 (2%)	15 (2.2%)	1.0
No	98 (98%)	675 (97.8%)	
Respiratory disorders			
Yes	9 (9%)	57 (8.3%)	
No	91 (91%)	633 (91.7%)	0.793
Chronic Kidney Disease			
Yes	1 (1%)	4 (0.6%)	
No	99 (99%)	686 (99.4%)	1.0
Others	4 (7.7%)	48 (92.3%)	0.268

**Table 2:** Knowledge, attitudes, and practices related to the influenza vaccination.

Characteristic	Regular Influenza Virus Vaccination (*N* = 100) *N* (%)	Irregular Influenza Virus Vaccination (*N* = 690) *N* (%)	*p*-Value
Aware of influenza symptoms			
Yes	88 (88.0)	573 (82.5)	
No	7 (7.0)	66 (9.5)	
Don’t know	5 (5.0)	51 (7.3)	0.453
Aware of influenza severity			
Yes	60 (60.0)	456 (65.7)	
No	23 (23.0)	138 (20)	
Don’t know	17 (17)	95 (13.7)	0.467
Aware of influenza risk			
Yes	61 (61)	368 (53)	
No	23 (23)	194 (28)	
Don’t know	16 (16)	128 (18.4)	0.352
Aware of vaccination necessity/availability			
Yes	93 (93)	571 (82.3)	
No	3 (3)	62 (8.9)	
Don’t know	4 (4)	57 (8.2)	0.031
Influenza vaccination is effective			
Yes	90 (90.0)	622 (90.0)	
No	10 (10.0)	69 (10.0)	1.0
The vaccine should be taken at a specific time			
Yes	55 (55)	275 (39.6)	
No	16 (16)	83 (12)	
Don’t know	29 (29)	332 (47.8)	0.002
The vaccine is safe			
Yes	93 (93.0)	616 (89.0)	
No	7 (7.0)	76 (11.0)	0.224
The vaccine is for children only			
Yes	7 (7)	50 (7.2)	
No	82 (82)	450 (64.8)	
Don’t know	11 (11)	190 (27.3)	0.001
Has a fear of needles			
Yes	8 (8)	36 (5.2)	
No	92 (92)	656 (94.5)	0.254
The vaccine is expensive			
Yes	-	8 (1.1)	
No	100 (100)	684 (98.6)	0.280
There is no need for the vaccine			
Yes	17 (17)	108 (16)	
No	83 (83)	583 (84.0)	0.725

**Table 3:** Sources of information about the influenza vaccine.

Resources	Regular Influenza Virus Vaccination (*N* = 100)	Irregular Influenza Virus Vaccination (*N* = 690)	*p*-Value
Pharmacist	3 (3.0)	27 (3.9)	0.659
Physician	20 (20)	147 (21.3)	0.776
Parents/family	36 (36)	296 (42.9)	0.402
Media	56 (56)	407 (59)	0.582
Government	4 (4)	48 (6.9)	0.268

**Table 4:** Multiple linear regression for the association between influenza vaccination history and participant perceptions.

Variable	β-Estimate	*p*-Value	95% Confidence Interval	
Thinks vaccination is safe	3.27	<0.001 *	2.067	5.171
Thinks vaccination is efficacious	2.87	<0.001 *	1.834	4.498
Believes the vaccination should be taken at a specific time	1.821	0.0059 *	1.188	2.789
Aware of influenza risks	1.334	0.1977	0.860	2.069
Aware of vaccination needs	2.781	0.0119 *	1.254	6.188
Aware of influenza symptoms	1.468	0.2426	0.771	2.797
Aware of influenza severity	0.788	0.2832	0.510	1.217

Note: * *p* < 0.05.

## Data Availability

The datasets generated and analyzed during the current study are not publicly available due to participant confidentiality but are available from the corresponding author on reasonable request.

## Appendix A. English Version of the Questionnaire

*I-Sociodemographic characteristics*

1. Gender: □ Male □ Female
2. Age: □ <24 years □ 24–33 years □ 34–51 years □ 52–64 years □ ≥65 years
3. Place of residence (Riyadh, Jeddah, Dammam…)
4. Education: □ No diploma □ Below high-school graduation □ High school diploma □ University degree
5. Employment: □ Yes □ No
6. Health Insurance: □ None □ Governmental/public issued □ Private
7. Participant’s perception of financial situation: □ Comfortable □ Manageable □ Difficult
8. How often do you smoke? □ Never □ <15 cigarettes daily □ 16–24 cigarettes daily □ > 25 cigarettes daily
9. Physical activity:
  □ Never exercise
  □ Exercise less than three times a week
  □ Exercise three times a week
  □ Exercise more than three times a week
10. Medical Visits
  □ Routinely
  □ Once a year
  □ When needed
11. Current medical history: Do you have any of the following chronic conditions?)
  	**Yes**	**No**	
  **Heart disease**			
  **Neurologic disease (epilepsy)**			
  **Diabetes Mellitus**			
  **Liver disease**			
  **Kidney disease**			
  **Cancer**			
  **Respiratory disorder (lung disease, COPD, asthma...)**			
  **Transplantation recipient**			
  **Guillain-Barre Syndrome**			
  **Anaphylactic allergies (swelling of mouth and difficulty breathing)**			
  **3 months or more of continuous steroid use**			
  **Other (please specify)**			
12. Medications: Are you currently taking any chronic medications?
  □ Yes
  □ No
  If yes, please specify:
  **Drug name (brand/generic name)**	**Indication (why do you use it?)**	**For how long have you been on it?**	**Do you take it regularly? Yes/No**
  II-Vaccination history:
13. Is your vaccination status up-to-date? □Yes □No
14. When was the last time you received a vaccine (Indicate year ex; 2009, 2014 or I don’t remember if patient cannot remember)?
  ________________________________________________________________________
15. How often do you receive the influenza vaccine? (ex: never, yearly, every 2 years...)
  ________________________________________________________________________
  III-Knowledge, attitude on influenza and vaccination
16. Knowledge of influenza infection and influenza vaccine
  	**Yes**	**No**	**I don’t know**
  **I am aware of the influenza symptoms**			
  **I am aware of the severity of influenza**			
  **I am aware of the patients at high risk from the influenza infection**			
  **I am aware there is a vaccine against influenza**			
  **The influenza vaccine is effective**			
  **The influenza vaccine should be taken during a specific time of the year (If answer is Yes, specify Month ex: Oct, Nov, Jan...)**			
  **The influenza vaccine is safe**			
  **The influenza vaccine is for children only**			
17. Are you willing to get vaccinated in general? □ Yes □ No
  If you answered no, please answer the below question:
  What is/are the reason(s) for not wanting to get vaccinated? (Choose all that apply):
  □ I don’t think the vaccines are effective□ I don’t think the vaccines are safe
  □ I don’t like to get injected (fear of needles)
  □ I don’t have time for vaccination
  □ It is difficult for me to access vaccines (costly)
  □ I believe vaccination is for children not for adults
  □ I don’t think there is a need to get vaccinated
18. Are you willing to get vaccinated with the influenza vaccine? □ Yes □ No
  If you answered no, please answer the below question:
  What is/are the reason(s) for not wanting to get vaccinated with the influenza vaccine? (Check all that apply):
  □ I don’t think the influenza vaccine is effective
  □ I don’t think the influenza vaccine is safe
  □ I don’t like to get injected (fear of needles)
  □ I don’t have time for vaccination
  □ It is difficult for me to access vaccine (costly)
  □ I believe influenza vaccination is for children not for adults
  □ I don’t think there is a need to get vaccinated
19. Where do you get information about vaccination?
  □ Doctor
  □ Pharmacist
  □ Religious leaders/organizations
  □ Media (TV/radio)
  □ Parents/friends
  □ Governmental agencies

Adapted from El Khoury G.M. et al. *Int. J. Environ. Res. Public Health* **2015**, 12, 15486–15497 [28].
